# Biogeography and Virulence of *Staphylococcus aureu*s

**DOI:** 10.1371/journal.pone.0006216

**Published:** 2009-07-13

**Authors:** Juan Fan, Min Shu, Ge Zhang, Wei Zhou, Yongmei Jiang, Yu Zhu, Guihua Chen, Sharon J. Peacock, Chaomin Wan, Wubin Pan, Edward J. Feil

**Affiliations:** 1 Department of Pediatrics, West China Second Hospital, Sichuan University, Chengdu, Sichuan, the People's Republic of China; 2 State Key Laboratory of Biotherapy and Cancer Center, West China Hospital, West China Medical School, Sichuan University, Chengdu, Sichuan, the People's Republic of China; 3 School of Pharmaceutical Sciences, Sun Yat-sen University, Guangzhou, People's Republic of China; 4 Crown Bioscience, Inc. (Beijing), Light Muller Building, ChangPing Science Park, Beijing, the People's Republic of China; 5 Department of Medicine, University of Cambridge, Addenbrooke's Hospital, Cambridge, United Kingdom; 6 Department of Biology and Biochemistry, University of Bath, Bath, United Kingdom; Institut Pasteur, France

## Abstract

**Background:**

*Staphylococcus aureus* is commonly carried asymptomatically in the human anterior nares and occasionally enters the bloodstream to cause invasive disease. Much of the global diversity of *S. aureus* remains uncharacterised, and is not clear how disease propensity varies between strains, and between host populations.

**Methodology:**

We compared 147 isolates recovered from five kindergartens in Chengdu, China, with 51 isolates contemporaneously recovered from cases of pediatric infection from the main hospital serving this community. The samples were characterised by MLST, the presence/absence of PVL, and antibiotic resistance profiling.

**Principal Findings:**

Genotype frequencies within individual kindergartens differ, but the sample recovered from cases of disease shows a general enrichment of certain MLST genotypes and PVL positive isolates. Genotypes under-represented in the disease sample tend to correspond to a single sequence cluster, and this cluster is more common in China than in other parts of the world.

**Conclusions/Significance:**

Virulence propensity likely reflects a synergy between variation in the core genome (MLST) and accessory genome (PVL). By combining evidence form biogeography and virulence we demonstrate the existence of a “native” clade in West China which has lowered virulence, possibility due to acquired host immunity.

## Introduction

*Staphylococcus aureus* is a significant human pathogen world-wide, particularly in health-care settings. The public health burden caused by this species is severely exacerbated by the widespread dissemination of clones resistant to *β*-lactam antibiotics (methicillin resistant *S. aureus*; MRSA) [1]. Resistance is conferred by an acquired penicillin-binding protein encoded by the *mecA* gene which is located on a large chromosomal element *SCCmec*. Whilst hospital-acquired (HA) MRSA strains tend to be characterised by large SCCmec elements corresponding to types I through III [2], isolates responsible for sporadic community-acquired (CA) disease, typically skin and soft-tissue infections (SSTIs) [3], [4], [5], harbour the smaller SCCmec cassette types IV through VIII [6], [7], [8], [9], [10], [11].

More severe CA-MRSA infection is often associated with specific toxins encoded by mobile elements [4]; the most notorious of these being the phage-encoded Panton-Valentine Leukocidin (PVL) toxin [12], [13]. PVL is an exotoxin which kills leukocytes by creating pores in the cell membrane. It is encoded by two co-transcribed genes, *lukF-*PV, and *lukS*-PV, which are currently known to be carried on four different phage [14], [15]. The leukotoxic action of PVL may lead to a high mortality rate associated with necrotizing pneumonia, even in young immunocompetent patients [16]. However, the danger posed to public health by a high frequency of PVL positive strains circulating in the community is currently unclear, as factors other than PVL may also contribute to the virulence of CA-MRSA [17], [18].

Given the increasing risks associated with community-acquired *S. aureus* infection, it is imperative to understand the population dynamics within the reservoir of asymptomatically carried clones from which these infections emerge. Detailed studies of the dissemination of virulence and drug-resistance determinants within the carriage population may inform on containment and intervention strategies for community infection. This is particularly pertinent in settings such as kindergartens, schools and army barracks where sporadic infections tend to be more common, yet little evidence exists on how the frequencies of potentially virulent clones may be amplified by localised transmission routes [19]. It is also remains unclear how clones circulating in the carriage population may differ in virulence potential, and whether such differences might result from transfer of mobile elements, variation within the core genome, or both.

Although the genotypes of HA-MRSA clones may differ markedly from both HA-MSSA isolates from the same hospitals or CA isolates [20], there is little evidence for differences between isolates causing sporadic CA infection and those circulating contemporaneously from healthy carriage in the same population [21]. Similarly, microarray analysis has also failed to detect any single genes associated with invasive disease [22]. This does not, however, necessarily imply that all strains are equally virulent. First, rapid changes in the accessory genome may not be reflected in the slowly evolving *S. aureus* core genome as assayed by MLST [23]. Second, the propensity to cause disease may be determined by subtle genetic changes or complex interactions between many core gene loci [24]. Host susceptibility and/or chance also play significant roles, as implicated by the wide range of symptoms noted during a recent study of a cluster of pediatric infections caused by a single strain [25].

Finally, asymptomatically carried samples remain under-represented in the current MLST datasets, particularly so for isolates from Africa [26] or mainland Asia [27]. These biases seriously undermine our ability to construct a comprehensive global framework of *S. aureus* clonal diversity, and to monitor the dissemination of drug resistance and virulence determinants. As well as representing important gaps in the global jigsaw, the relative paucity of molecular data for Africa and mainland Asia has direct public health implications for these regions. Here we address these issues by characterising and comparing isolates from healthy children from five separate kindergartens and from cases of pediatric infection in Chengdu, China. Although there have been previous studies on pediatric *S. aureus* carriage in Taiwan [28], [29], to our knowledge this is the first such study carried out in mainland China, and the first to compare contemporaneous samples from pediatric infection and carriage.

## Results

### Antimicrobial resistance profiles

A total of 801 children were enrolled in the study of nasal carriage. Of these, 147 (18.35%) were asymptomatically colonized with *S. aureus*. The carriage rate within kindergartens ranged from 13.8% (kindergarten E) to 22.8% (kindergarten C). The full resistance profiles of the carriage strains are given in Table S1, and summarised by kindergarten in Table 1. Nine of the carriage isolates were CA-MRSA (with oxacillin MIC≥4 mg/L), corresponding to 6.1% of all carriage isolates and 1.1% of all children. These isolates show varying resistance patterns to the other antibiotics. All the 9 CA-MRSA were resistant to Pen, 7/9 (78%) resistant to Ery, 2/9 (22%) resistant to Cli and 1/9 (11%) resistant to Rif. Overall, resistance was noted at the following frequencies: Oxa (6.1%), Pen (91.8%), Ery (81.6%), Cli (39.5%), Rif (2%), which are similar (but slightly lower) frequencies than for the disease isolates (see below). No isolates were vancomycin resistant, and there were no obvious differences when the resistance data are broken down according to kindergarten.

**Table 1:** 

Kindergarten	OX(%)	PEN(%)	ERY(%)	CLI(%)	RIF (%)	PVL(%)	N	Carriage Rate (%)
A	2(4.9)	38(92.7)	35(85.4)	16(39)	0(0)	9(22)	41	19.43
B	2(10)	19(95)	17(85)	5(25)	0(0)	3(15)	20	17.39
C	1(3.6)	25(89.3)	24(85.7)	16(57.1)	1(3.6)	7(25)	28	22.76
D	1(4.2)	21(87.5)	19(79.2)	10(41.6)	1(4.2)	5(20.8)	24	22.64
E	3(8.8)	32(94.1)	25(73.5)	11(32.4)	1(2.9)	9(26.5)	34	13.82
TOTAL	**9(6.1)**	**135(91.8)**	**120(81.6)**	**58(39.5)**	**3(2)**	**33(22.4)**	**147**	**18.35**

The full resistance data for the 51 isolates from cases of pediatric infection are given in Table S2, and summarised according to clinical source in Table 2. Of these 51 disease isolates, 40 (78%) were classified as community-acquired on the basis that they were isolated<48 hours after admission, and that the patient had not been hospitalised within the preceding 12 months (Table S2). A total of 10 isolates (19.6%) were MRSA; seven of these were CA, three were HA. Eight of the 10 MRSA isolates were recovered from sputum; otherwise there was no clear pattern of drug resistance according to clinical source. Overall, resistance was noted at the following frequencies: Oxa (19.6%), Pen (98%), Ery (86.3%), Cli (45.1%) and Rif (3.9%). None of the disease isolates were resistant to vancomycin.

**Table 2:** 

Clincal Source	OX(%)	PEN(%)	ERY(%)	CLI(%)	RIF(%)	PVL(%)	N
Blood	1(11.1)	9(100)	7(77.8)	2(22.2)	0(0)	6(66.7)	9
Pharynx	1(14.3)	7(100)	7(100)	5(71.4)	0(0)	5(71.4)	7
Pus	0(0)	9(90)	9(90)	6(60)	0(0)	10(100)	10
Sputum	8(36.4)	22(100)	20(90.9)	10(45.5)	2(9.1)	18(81.8)	22
Other	0(0)	3(100)	1(33.3.)	0(0)	0(0)	2(66.7)	3
**TOTAL**	**10(19.6)**	**50(98)**	**44(86.3)**	**23(45.1)**	**2(3.9)**	**41(80.4)**	**51**

### The presence of PVL

Thirty-three of the carriage isolates (22.4%) were positive for the PVL gene, including 5 of the 9 CA-MRSA isolates (Table 1). In contrast, 41 of the disease isolates (80.4%) were positive for the PVL gene, including 6/10 of the MRSA isolates, and 9/11 (81%) of the HA isolates (Table 2). A two-by-two chi-sq. test [(33, 114), (41, 10)] confirmed that the frequency of PVL from cases of infection was significantly higher than the carriage sample (χ^2^ = 54.3, P<0.0005).

### Multilocus Sequence Typing (MLST)

#### i) Carriage isolates

Of the 147 isolates from the pooled carriage sample, 26 STs were observed, 5 of which were novel (Table S1). The most frequent clonal complex was CC121, which accounted for 50/147 (34%) of the carriage sample. Also of note is the relatively high frequency (12/147; 8%) of ST398; in Europe this genotype is frequently associated with farm animals, but is very rarely isolated from humans [30]. One genotype, ST942, was observed in four isolates. This sequence type does not correspond to any of the currently recognised clonal complexes, although it was recently detected in a carriage sample from Switzerland [31], and is a DLV of ST707 which has been recorded in Poland and Canada (http://saureus.mlst.net/). Five novel clonal variants were observed in the carriage sample: ST941 (SLV of ST15), ST940 (SLV of ST59), ST946 (DLV of ST121), ST945 (SLV of ST182) and ST943 (SLV of ST7). We found no clear differences in genotype frequency corresponding to the age of the carrier.

#### ii Disease isolates

The 51 isolates from cases of pediatric infection defined 20 STs, 8 of which were absent from the carriage sample (Table S2). Thus, for both samples combined, a total of 34 genotypes were noted, but only 12 of these were common to both carriage and disease isolates. Of the 8 genotypes only noted amongst disease, 5 were novel. These novel STs were clonal variants of known founders, corresponding to ST947 (DLV of ST121), ST948 (SLV of ST6), ST949 (DLV of ST6), ST950 (SLV of ST5), ST951 (SLV of ST59). Of these, only ST948 was classified as hospital-acquired. As for the carriage sample, the most frequent clonal complex in the disease sample was CC121 (17.6%; ST121, n = 8, ST947, n = 1).

#### iii MRSA isolates

Of the nine MRSA isolates present in the carriage data, 6 corresponded to ST59. This is consistent with the study of Lo et al who noted that ST59 accounted for all nine of the CA-MRSA isolates recovered from pediatric carriage from a kindergarten in Taiwan [28]. The other 3 MRSA in the carriage sample corresponded to single isolates of STs 398, 30 and 942. In contrast, the ten MRSA from the disease sample corresponded to a broad range of genotypes (STs 5, 20, 88, 121, 188, 573 and 623 from CA isolates, and STs 39, 88 and 239 from HA isolates). We note that ST59 was not represented in MRSA from disease (MRSA isolates shown in blue in Table S1).

#### iv Comparisons to the MLST database and previous carriage studies

**Figure 1:**
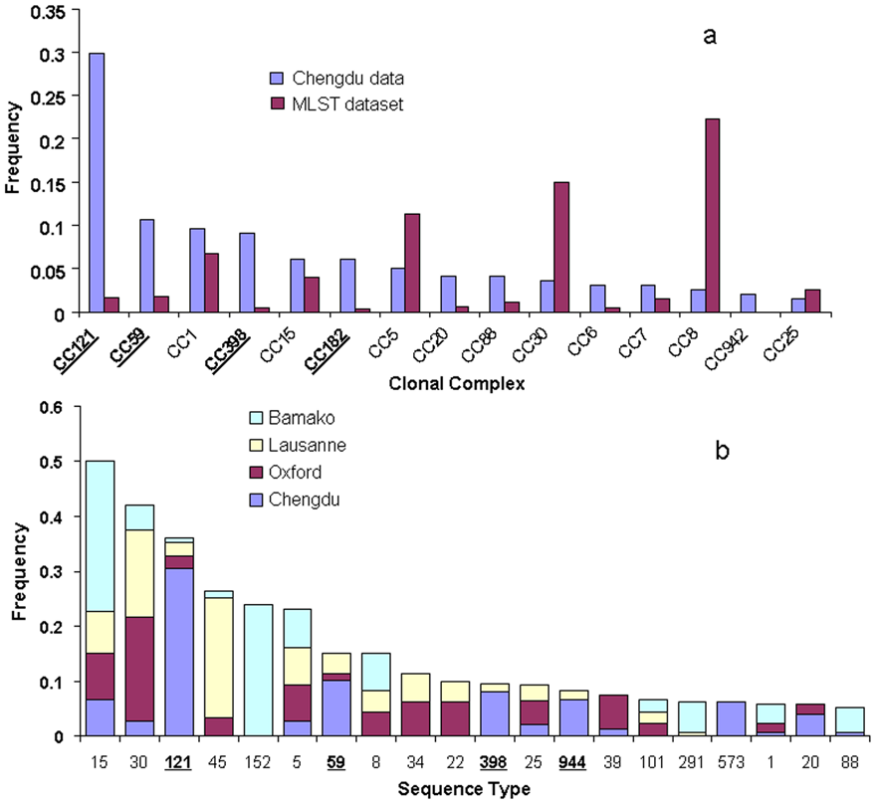
a The frequencies of the clonal complexes in the combined disease and carriage dataset, and the corresponding frequencies in the MLST website are given. The clonal complexes were delineated using eBURST on the Chengdu data separately (198 isolates) and on the whole MLST dataset. eBURST also outputs the number of isolates belonging to the complex which was used to calculate the frequencies. CCs121, 59, 398 and 182 are all over-represented in the Chengdu data (in bold and underlined). These complexes are related, and all correspond to the major phylogenetic clade Group 1b (see text). In contrast, CCs 5 and 8 are under-represented in the Chengdu data (Group 2), as is CC30 (Group 1a). 1b Comparison of the frequencies of STs within the Chengdu carriage isolates with previous studies of carriage in Europe and Africa (see text for references). Only STs with an average frequency of>1% over the four datasets were included. These comparisons confirm the atypical genotype frequencies noted above (note: ST944 = CC182, ST573 = CC1).

**Figure 2:**
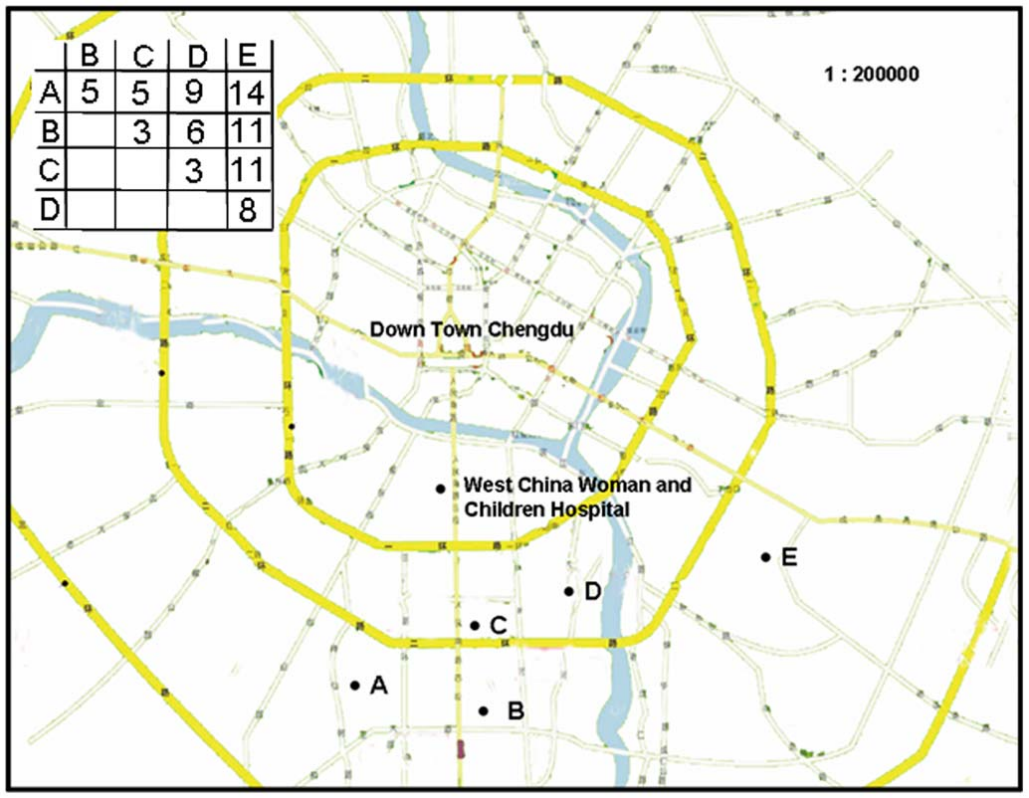
Map showing the locations of the West China Woman and Children Hospital (where the invasive samples were recovered), and five kindergartens A, B, C, D and E, where carriage samples were recovered. The inset shows the distances (in Km) between the kindergartens.

Although almost all disease and carriage isolates corresponded to known clonal complexes by MLST, the frequencies of the clonal complexes in the Chengdu data are markedly different from those observed in the MLST database, which is composed of a mixture of carriage and disease isolates mostly derived from Europe, Australia, and the USA. eBURST was used to delineate clonal complexes (http://saureus.mlst.net/eburst/; [32]), and Figure 1a shows frequencies of clonal complexes in the pooled Chinese data (infection plus all carriage samples) and the corresponding frequencies in the MLST database. Although CC121 accounts for almost 30% of the current combined dataset, it only accounts for<2% of the isolates in the MLST database. CCs 59, 182 (STs 944, 945) and 398 are also observed surprisingly frequently, given their frequencies in the MLST database (underlined in Figure 2). In contrast, complexes CCs 30 and 8 are observed less frequently than expected given their frequencies in the MLST database.

A potential problem with this comparison lies in the fact that the MLST database is not a representative population sample, but was assembled piecemeal from different studies aimed at addressing questions of local epidemiology in different regions of the world. Furthermore, the data uploaded onto the MLST database frequently only contains one example of each ST, or only new STs, from a given study. In order to address these biases, we also compared the carriage data from the current study with comparable studies on carriage in Europe (Oxford, UK [21] and Lausanne, Switzerland [33]), and Africa (Bamako, Mali [26]). As many STs were found rarely in only one or two of the datasets, only those STs with an average frequency of>1% (over the four datasets) were compared. This analysis confirmed the over-representation of the STs 121, 59, 398 and 944 (CC182) in the Chengdu carriage population compared to the other samples (Figure 1b).

#### v Localised clonal expansions within the carriage population

**Figure 3:**
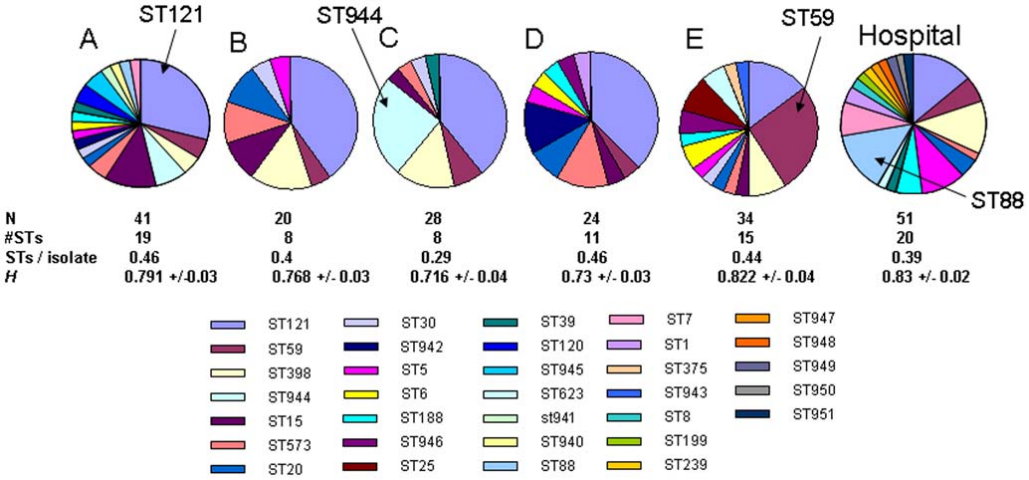
MLST genotypes of the carriage data broken down according to kindergarten. Two possible examples of local clonal spread are indicated by arrows: i) the high frequency of ST59 in kindergarten E, and ii) the high frequency of ST944 in kindergarten C. The diversity within each kindergarten was highly consistent, except for kindergarten C which shows a lower number of STs per isolate (not significant p<0.05), and the lowest average heterozygosity per locus (H), calculated using Lian v3.5 as implemented on www.pubmlst.org. The breakdown for the disease sample is also shown for comparison, and reveals a high frequency of ST88.

Figure 2 shows the location of the five kindergartens from which the carriage isolates were sampled, and the distance matrix (in Km) between them (inset). Figure 3 gives a breakdown of the MLST genotypes in the carriage sample according to kindergarten (A–E). ST121 is the dominant clone in kindergartens A–D, but ST59 is more common in kindergarten E, corresponding to 9/34 isolates (26.5%) compared to 5/34 (14.7% for ST121). This is consistent with the clonal spread of ST59 due to localised transmission within this kindergarten. A second example of local clonal spread is the high frequency of ST944 in kindergarten C (7/28 isolates; 25%). ST944 is only noted in one other kindergarten (A), and at a much lower frequency (3/41; 7.3%). A resampling procedure revealed that the high frequency of ST944 in kindergarten C was statistically significant (P<0.001; data not shown). For comparison, Figure 3 also shows the proportion of STs within the disease sample. This sample is most notable for high proportion of ST88, as discussed below. For kindergartens A, B, D, E, and the disease sample, the number of STs per isolate occupied a narrow range (0.39–0.46), consistent with a linear increase in the number of unique STs with sample size [34]. However, for kindergarten C the corresponding figure was 0.29, and the average heterozygosity per locus (H) was also lowest for this kindergarten, possibly resulting from the clonal spread of ST944.

#### vi Comparison of disease and carriage data

**Figure 4:**
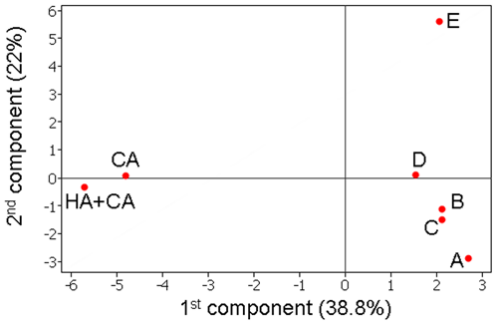
Principal components analysis. The datasets from the five kindergartens (A–E), the total disease dataset (CA+HA) and just the community-acquired dataset (CA) were plotted according to the two largest components. These accounted for 38.8% and 22% of the variation in the data respectively. The first component separated the disease isolates from the carriage isolates, whilst the second component distinguished between the different kindergarten samples.

Given the evidence for clonal expansion within individual kindergartens, we used principal component analysis to compare genotype frequencies between the pooled carriage samples and the disease sample. Figure 4 plots each dataset according to the first two components, which account for 38.8% and 22% of the variation in the data respectively. To check for bias caused by the small number of HA isolates in the disease sample, and the slightly larger size of the disease sample, we also included a dataset consisting of only the 40 community-acquired disease isolates. The first component clearly separates the disease and carriage samples, even when the HA isolates are excluded. The second component separates kindergarten E, which is consistent with its physical location, being on average 11 Km from the other kindergartens (compared to 8.25, 6.25, 5.5 and 6.5 for kindergartens A–D respectively; Figure 2).

**Figure 5:**
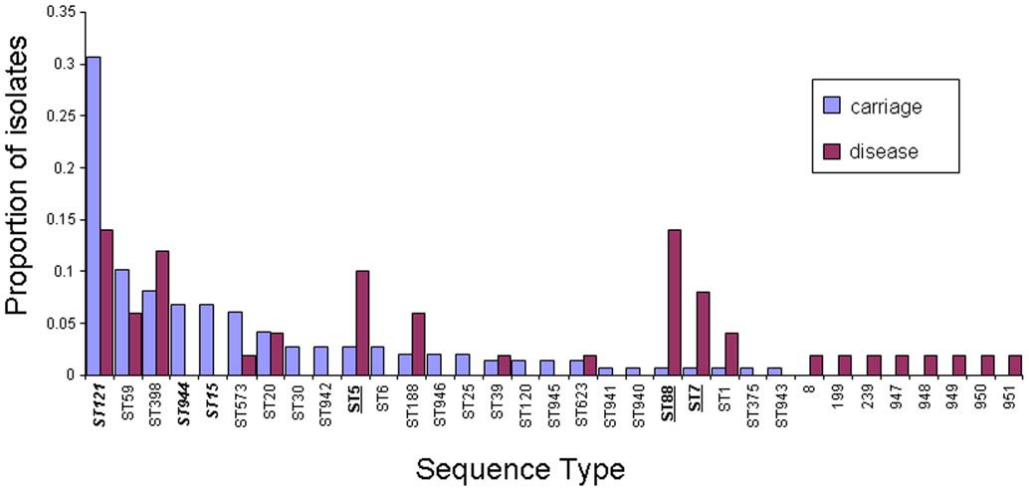
The proportions of each ST noted in the disease and pooled carriage sample. A resampling procedure showed that STs 121, 944 and 15 were significantly under-represented from cases of disease (bold, italics), whilst STs 5, 88 and 7 were significantly over-represented from cases of disease (bold, underlined).

Having confirmed the distinctiveness of the disease sample, we pooled the 147 isolates from the five kindergartens and compared the frequencies of the genotypes of all carriage isolates with the 51 disease isolates. Figure 5 shows the proportion of every ST recorded in the two datasets, and we tested for significant differences between disease and carriage using the re-sampling procedure described in Methods.

Three sequence types (STs 121, 944 and 15) were significantly under-represented in the disease sample, whilst three STs were over-represented (STs 5, 88 and 7) which is consistent with heightened virulence of these genotypes. As noted above, the high frequency of ST88 in the disease sample (7/51; 13.7%) was striking, given that only one ST88 isolate was present in the carriage sample (0.7%). Five of the 7 ST88 disease isolates were community-acquired. We find no evidence that the differences in disease potential are simply a reflection of the frequency of PVL within the STs. For example, ST121 exhibits a PVL frequency slightly higher than the average for the carriage data (26%), yet this genotype is significantly under-represented in cases of disease.

#### vii Phylogenetic analysis

**Figure 6:**
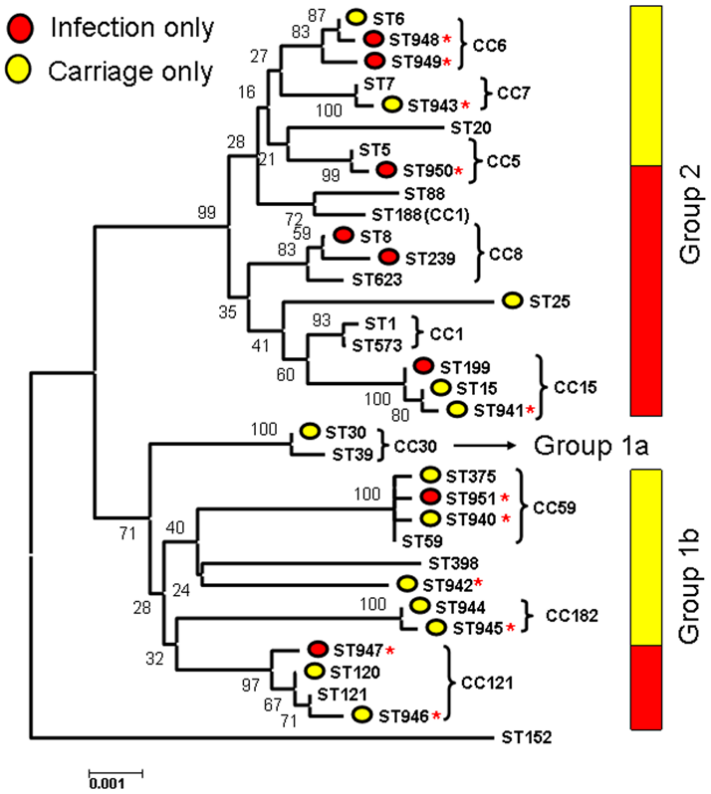
A neighbour-joining tree based on the concatenated sequences of each of the 32 sequence types noted in the combined dataset, as implemented in Mega v4.0 [46] using Kimura-2-parameter distances. Bootstrap support (based on 1,000 replicates) are given on the nodes. The most basal nodes are reasonably well supported and correspond to the delineation of the three major groups (1a, 1b and 2) identified previously [35]. The grouping of STs into CCs (terminal clusters) is shown by curly brackets. Note the inconsistent position of ST188 (Group 2; CC1), which is a DLV of ST1 but clusters with ST88 on the basis of allele sequences due to recombination. STs which were only noted in the disease sample are marked by a red circle, whilst STs only noted in the carriage sample are marked by a yellow circle. STs common to both datasets are not marked with a circle. Novel STs (first noted in the current study) are marked by a red asterisk. The proportion of *isolates* corresponding to the disease and carriage datasets are shown by red and yellow bars for Groups 2 and 1b. There is a significantly higher proportion of disease isolates within Group 2.

**Figure 7:**
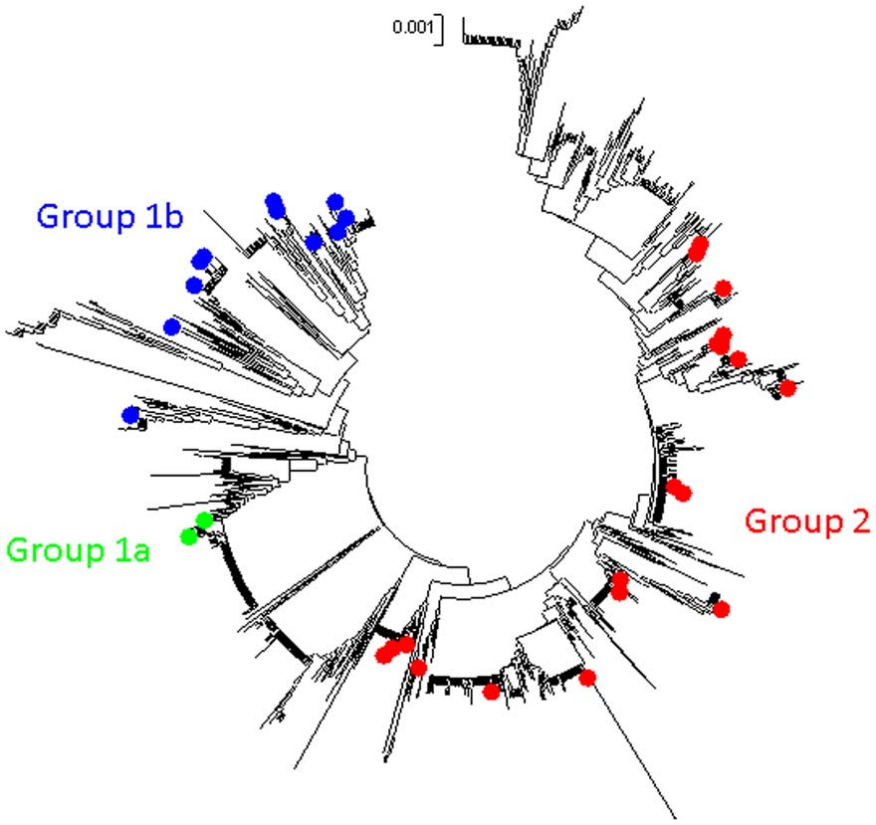
A neighbour-joining tree of 1294 STs from the MLST database with the STs in the Chengdu datasets highlighted. This confirms the cohesion of these three groups within the broad context of *S. aureus* diversity.

A neighbour-joining tree based on the concatenated MLST data of all the carriage and infection isolates is given in Figure 6. The divergent genotype ST 152 [26] was included as an outgroup. Two major clades are resolved as noted previously from MLST data [21], [26] and from using a Bayesian approach on based on data from 40 unlinked loci [35]. We also confirmed that these groups remained coherent within the broad context of *S. aureus* diversity, by reconstructing a NJ tree based on 1294 concatenated STs from the MLST database (Figure 7). Group 2 (upper clade; CCs 1, 5, 6, 7, 8, 15, 88, 188 & 623) is well supported by the current data (bootstrap score of 99). The lower clade has previously been divided in to two groups (1a and 1b) [35] but in the current study Group 1a is only represented by 7 isolates of CC30. Group 1b consists of CCs 121, 59, 182 [ST944 and ST945], 398 and the newly discovered ST942.

We note that Group1b corresponds to those clonal complexes present at atypically high frequencies in the Chengdu population (Figures 1a, 1b). Thus the current dataset is characterised by a high frequency of lineages which are rarely recovered elsewhere, and which appear to be related. Figure 5 points to a second surprising feature of these data; those 3 STs over-represented in the disease sample correspond to Group 2, whilst those 3 STs under-represented from cases of disease correspond to Group 1b. The only exception is ST15 which is under-represented (absent) from the disease sample yet corresponds to Group 2. To examine this further, we tested whether there was a significant difference in the total number of disease and carriage isolates corresponding to Groups 2 and 1b (excluding the 7 Group 1a isolates). Group 2 accounts for 30/49 (61.2%) of the disease isolates but only 46/141 (32.6%) of the carriage isolates. In contrast, 67.4% of the carriage isolates and 38.8% of the disease isolates correspond to Group 1b. These proportions are given by the red and yellow bars in Figure 5. A two-by two chi-sq test [(30, 19), (46, 95)] showed this difference to be highly significant (P<0.005; Figure 5).

In sum, these data therefore point to the following: i) Group 1b isolates are much more common in West China than in other parts of the World, and ii) Group 1b isolates are also significantly less likely to be associated with disease in the study population than Group 2 isolates.

## Discussion

Here we present MLST data from five contemporaneous samples of carried *S. aureus* isolates from kindergartens in Chengdu, China, and a single sample from cases of pediatric disease from the main hospital serving the same community. We draw comparisons between the individual carriage samples, and between the pooled carriage sample and the disease sample. We also exploit the portability of MLST data by comparing the pooled Chengdu dataset (carriage plus disease) with the entire MLST dataset for this species, and with comparable studies of local carriage populations. This study therefore encompasses geographical differences in the carriage population at a local and global level, in addition to differences in the propensity to cause community-acquired disease. By addressing these questions simultaneously we were able to detect a major clade of related clonal lineages which are i) relatively rarely observed in other continents, and ii) show a lower propensity to cause disease within the study population.

A possible source of sampling bias in this study rests on the assumption that the carriage sample is representative of the community from which the disease cases derived. Great care was taken to minimise these biases. Disease and carriage isolates were recovered contemporaneously, and we carefully checked for biases due to patient gender or age. Furthermore, the patients all lived within the catchment area of the hospital where the disease sample was recovered and the majority of the disease isolates could be confidently assigned as community-acquired. We are confident that the inclusion of a small number of hospital-acquired isolates does not account for the differences in the disease sample. This is supported both by the principal component analysis, and the observation that two of the three STs over-represented in disease were absent amongst the HA isolates (ST7 and ST5), and 5/7 ST88 isolates were found amongst the CA isolates. Finally, single local expansion events in the community should not result in a serious bias in the disease sample, as these cases represent a wide catchment area. Nevertheless, we add the important caveat that the sample sizes of both the carriage and disease datasets are not sufficiently large to unequivocally rule out chance effects, and future studies in Chengdu or other regions of South West China are required to confirm these apparent differences.

Given previous failures to detect significant differences between disease and carriage samples on the basis of MLST data [21], [22], it is surprising that STs 88, 7 and 5 appear to be enriched in the disease sample. ST88 is the most convincing case, accounting for 14% of the disease isolates and>1% of the carriage isolates. Yu et al noted ST88 to be the most common clone among PVL positive isolates recovered from hospitalised patients in Wenzou, in the South East of China [36]. Widespread acquisition of resistance by ST88 may therefore constitute a significant public health burden, and close monitoring is required. It is also noteworthy that CC121 accounts for approximately one third of the carriage population, double the frequency observed in the disease sample.

We find a highly significant enrichment for PVL positive isolates (80.4%) within the disease sample. This is also difficult to explain by a systematic sampling bias, as PVL was distributed reasonably consistently throughout strains from different kindergartens (15%–26.5%). This observation therefore strongly supports the view that the presence of PVL impacts on the propensity to cause serious pediatric infection. Importantly, the distribution of PVL between MLST genotypes does not appear to explain the under- or over- representation of certain STs within the disease sample, nor the differing disease potential at a broad phylogenetic scale (between major clades). We consider it likely that synergistic effects between the core and accessory genomes are important; that is to say that PVL may increase disease potential, but the magnitude of this effect is dependent upon the clonal background. If so, assays on the frequency of PVL positive isolates in the carriage population may not provide entirely accurate risk assessments in the absence of a population framework.

The frequencies of PVL detected in our samples are much higher than recent data for elsewhere in China. Yu et al recently noted a PVL frequency of 11.9% among 160 HA isolates and 17.1% of 35 CA isolates recovered from patients presenting at the First Affiliated Hospital of Wenzhou Medical College [36]. Lo et al recently noted a 19.1% prevalence of PVL positive strains among MRSA isolates recovered from pediatric carriage in Taiwan, and concluded that previous antibiotic use is a major risk factor for carriage of PVL positive isolates [29].

In addition to examining differences in disease potential, this study also reveals striking biogeographical trends within the carriage data at a local and global level. Of the five kindergartens sampled, two showed predominance of a single clone which is not reflected in carriage data as a whole (ST944 in kindergarten C, and ST 59 in kindergarten E). These trends are consistent with clonal expansion owing to localised transmission, as predicted by the neutral epidemic model [19], and highlight the role of settings such as kindergartens for the rapid dissemination of specific clones.

At the other end of the scale, comparisons between the Chengdu data and the entire MLST database and previous carriage datasets reveal atypically high frequencies of CCs 121, 59, 398 and 182, and atypically low frequencies of CCs 5, 8 and 30 within the study population. Of particular note is CC121 which accounts for almost 30% of the Chengdu isolates but<2% of isolates from other regions. Whilst it is not in itself surprising to observe atypical genotype frequencies within a single region for which there is currently little data, it was not forseen that all atypically common lineages would correspond to a single major clade (Group 1b). It is tempting to speculate that Group 1b represents the “native” carriage population for China and possibly other Asian countries, and the extant pattern is a footprint from times preceding mass global migration. If so, this pattern should shed light on the long-term evolution and dissemination of *S. aureus*. We note that geographical structuring has recently been observed within a single sequence type using SNP discovery [37] but this very fine-scaled analysis differs from the current study in that informs on more recent clonal dissemination.

Finally, in addition to being more common in China than in other parts of the world, Group 1b isolates were also significantly less likely to be recovered from cases of disease than group 2 isolates. Together these observations may point to the role for host immunity in providing protection for the most commonly encountered genotypes. This interpretation should be treated with caution, however, as we cannot rule out the possibility of the chance clustering of the low virulent genotypes within a single cluster. Further, MRSA isolates of the group 1b complexes CCs121 and 59 have been recovered from cases of disease in east Asia. For example, ST59 is common amongst PVL positive CA-MRSA isolates in Taiwan, and is thought to have clonally spread in this country [38], [39], where it is particularly associated with pediatric infection [40]. Despite these caveats, our analysis indicates that a full understanding of virulence potential in human pathogens requires both phylogenetic and biogeographic contexts.

## Materials and Methods

### Nasal carrier isolates

Eight hundred and one children, age from 2 to 7 years from 5 kindergartens in Chengdu, Sichuan province, China, were enrolled in the study from September to December, 2005. The locations of the five kindergartens in Chengdu are shown in Figure 2, and a matrix of distances between them (in Km) shown in the inset. Among the 801 children, 26.3% (211/801) were from kindergarten A, 14.4% (115/801) from kindergarten B, 15.4% (123/801) from kindergarten C, 13.2% (106/801) from kindergarten D and 30.7% (246/801) from kindergarten E; 22.3% (179/801) (Table S1; Table 1). The age distribution of the children was as follows: 2 to 3 yrs, 18.9% (151/801); 3 to 4 yrs, 24.1% (193/801); 4 to 5 yrs, 18.0% (144/801), 5 to 6 yrs, 16.7% (134/801); 6 to 7 yrs, 22.3% (179/801). There was a slight predominance of boys (414, 51.7%). Sample collection protocol for the study was approved by the medical ethical committee of West China Secondary Hospital, consent letters were signed by parents or guardians before sample collection and questionnaires for medical and family information were administered.

Nasal swabs were taken with dry cotton swabs from enrolled children from September to December, 2005 and immediately placed in the sterile culture tube. Each nasal swab was then plated respectively onto mannitol-salt agar directly within 4 hours of collection. After incubation at 37°C in air for 24 hours, yellow colonies were examined by Gram staining and using a Slidex Staph Plus (bioMerieux, SA, Marcy I'Etoile, France) to confirm the identification of *Staphylococcus aureus*. ATCC29213 was included as a control.

### Disease isolates

Fifty-one non-duplicate isolates were recovered from *S. aureus* infections in children aged between 2 weeks and 14 years at West China Woman and Children's hospital, Chengdu from January 2004 to April 2006 (Table S2). The sites of isolation were as follows: respiratory secretions (22/51; 43.1%), blood-stream (9/51; 17.7%), pus (10/51; 19.6%), pharyngeal swab (7/51; 13.7%), synovial fluid (1/51; 2%), stool (1/51; 2%) and conjunctival exudate (1/51; 2%). The clinical symptoms associated with these isolates are given in Table S2. The single isolate recovered from stool (17S) was associated with staphylococcal food poisoning. Of the isolates recovered from respiratory secretions, 20/22 samples were obtained during bronchoscopy performed on patients with clinical and radiological features of pneumonia, in whom the bronchoscopic findings were consistent with pneumonia. The remaining two cases were children with pneumonia and bronchitis in whom the diagnosis was made by a combination of clinical features and chest x-ray. Of the isolates recovered from the pharynx, isolate 365S was recovered from a patient with tonsillitis and Staphylococcal Scarlet Fever (SSF), whereas isolates 2215, 704S, 487S and 905S were recovered from pus from the tonsils of patients diagnosed with suppurative tonsillitis. Two further isolates recovered from the pharynx, 315S and 653S, were from patients diagnosed with Staphylococcal scalded skin syndrome (SSSS). The gender and age (in months) of each patient is also given in Table S2.

Isolates were defined as community-acquired (CA) if they were identified within 48 hours of hospitalization from a child with features consistent with *S. aureus* infection who had not been hospitalized in the preceding 12 months. Isolates recovered>48 hours after admission in patients with an unrelated diagnosis were categorized as hospital-acquired (HA). *S. aureus* isolates with oxacillin MIC≥4 mg/L are defined as MRSA (methicillin-resistant *S. aureus*).

The sampling strategy was carefully designed so as to validate a case control study through comparisons of disease and carriage isolates. The carrier and disease isolates were collected contemporaneously, and the disease samples were taken from pediatric patients at West China Children's hospital, which is the hospital serving the population from which the carriage sample was drawn.

### Antibiotic susceptibility tests

Antibiotic susceptibility testing was performed using Bauer-Kirby method [41] on Mueller-Hinton medium for preliminary screening and then results further confirmed by the agar dilution method in accordance with the CLSI recommendations to determine the minimal growth-inhibitory concentrations (MICs) of all recovered isolates against six antibiotics: oxacillin, erythromycin, penicillin, vancomycin, rifampicin and clindamycin (Sigma Chemical Co., Ltd., St. Louis, Mo.). ATCC29213 (MSSA) and ATCC 43300 (MRSA) were used throughout as controls.

### PVL assay

The bacterial chromosomal DNAs were extracted from cells that had been cultured overnight by using a genomic DNA preparation kit (Tiangen, China) with lysostaphin at 10 mg/ml and RNaseA at 25 mg/ml for the lysis step. All isolates were assayed for the presence of PVL genes by PCR using the *luk-PV1* and *luk-PV2* primers as previously described [42].

### Mulitlocus Sequence Typing (MLST)

For multilocus sequence typing (MLST), PCR products were obtained using primers and protocols described previously [43]. DNA sequencing was performed by the Beijing Genomics Institute of Chinese Academy of Sciences, using the ABI 3730 DNA sequencer with ABI Dye terminator cycle sequencing kit (BigDye v3.1, Foster City, CA, USA). Sequences were then submitted to the MLST database (http://www.mlst.net) for the generation of an allelic profile and sequence type (ST). MRSA genotypes were defined based on MLST and SCC*mec* types, as proposed by Robinson and Enright [44]. Clustering patterns between the isolates were examined using eBURST (http://saureus.mlst.net/eburst/; [32]) and START (http://pubmlst.org/software/analysis/start/; [45]). The concatenated MLST data was downloaded from http://saureus.mlst.net/and a neighbour-joining tree was reconstructed using the Kimura-2-parameter distance measure as implemented in MEGA v. 4.1 [46]. Heterozygosity was calculated using Lian v 3.5 [47] as implemented on http://www.pubmlst.org.

### Comparing the disease and carriage datasets

In order to examine differences in MLST genotype frequencies between the disease sample and the five carriage samples, we used two approaches. First, we used principal components analysis (PCA) on genotype frequencies. Second, we compared the genotype frequencies within the disease sample with the pooled carriage sample by drawing 1000 random samples of 51 isolates (with replacement) from the 147 carriage isolates and comparing the frequency of each clonal complex in these random samples with that observed in the disease sample. Significance was gauged by calculating the 5^th^ and 95^th^ percentiles from the 1000 resampled datasets for each complex.

## Supporting Information

Table S1 (0.05 MB XLS)

Table S2 (0.03 MB XLS)

## References

[1] Enright MC, Robinson DA, Randle G, Feil EJ, Grundmann H (2002). The evolutionary history of methicillin-resistant Staphylococcus aureus (MRSA).. Proc Natl Acad Sci U S A.

[2] Ito T, Katayama Y, Asada K, Mori N, Tsutsumimoto K (2001). Structural comparison of three types of staphylococcal cassette chromosome mec integrated in the chromosome in methicillin-resistant Staphylococcus aureus.. Antimicrob Agents Chemother.

[3] Said-Salim B, Mathema B, Kreiswirth BN (2003). Community-acquired methicillin-resistant Staphylococcus aureus: an emerging pathogen.. Infect Control Hosp Epidemiol.

[4] Paez A, Skiest D (2008). Methicillin-resistant Staphylococcus aureus: From the Hospital to the Community.. Curr Infect Dis Rep.

[5] Nygaard TK, DeLeo FR, Voyich JM (2008). Community-associated methicillin-resistant Staphylococcus aureus skin infections: advances toward identifying the key virulence factors.. Curr Opin Infect Dis.

[6] Deurenberg RH, Vink C, Kalenic S, Friedrich AW, Bruggeman CA (2007). The molecular evolution of methicillin-resistant Staphylococcus aureus.. Clin Microbiol Infect.

[7] Zhang K, McClure JA, Elsayed S, Conly JM (2009). Novel staphylococcal cassette chromosome mec type, tentatively designated type VIII, harboring class A mec and type 4 ccr gene complexes in a Canadian epidemic strain of methicillin-resistant Staphylococcus aureus.. Antimicrob Agents Chemother.

[8] Ma XX, Ito T, Tiensasitorn C, Jamklang M, Chongtrakool P (2002). Novel type of staphylococcal cassette chromosome mec identified in community-acquired methicillin-resistant Staphylococcus aureus strains.. Antimicrob Agents Chemother.

[9] Ito T, Ma XX, Takeuchi F, Okuma K, Yuzawa H (2004). Novel type V staphylococcal cassette chromosome mec driven by a novel cassette chromosome recombinase, ccrC.. Antimicrob Agents Chemother.

[10] Oliveira DC, Milheirico C, de Lencastre H (2006). Redefining a structural variant of staphylococcal cassette chromosome mec, SCCmec type VI.. Antimicrob Agents Chemother.

[11] Berglund C, Ito T, Ikeda M, Ma XX, Soderquist B (2008). Novel type of staphylococcal cassette chromosome mec in a methicillin-resistant Staphylococcus aureus strain isolated in Sweden.. Antimicrob Agents Chemother.

[12] Vandenesch F, Naimi T, Enright MC, Lina G, Nimmo GR (2003). Community-acquired methicillin-resistant Staphylococcus aureus carrying Panton-Valentine leukocidin genes: worldwide emergence.. Emerg Infect Dis.

[13] Martinez-Aguilar G, Avalos-Mishaan A, Hulten K, Hammerman W, Mason EO (2004). Community-acquired, methicillin-resistant and methicillin-susceptible Staphylococcus aureus musculoskeletal infections in children.. Pediatr Infect Dis J.

[14] Ma XX, Ito T, Chongtrakool P, Hiramatsu K (2006). Predominance of clones carrying Panton-Valentine leukocidin genes among methicillin-resistant Staphylococcus aureus strains isolated in Japanese hospitals from 1979 to 1985.. J Clin Microbiol.

[15] Feng Y, Chen CJ, Su LH, Hu S, Yu J (2008). Evolution and pathogenesis of Staphylococcus aureus: lessons learned from genotyping and comparative genomics.. FEMS Microbiol Rev.

[16] Gillet Y, Issartel B, Vanhems P, Fournet JC, Lina G (2002). Association between Staphylococcus aureus strains carrying gene for Panton-Valentine leukocidin and highly lethal necrotising pneumonia in young immunocompetent patients.. Lancet.

[17] Voyich JM, Otto M, Mathema B, Braughton KR, Whitney AR (2006). Is Panton-Valentine leukocidin the major virulence determinant in community-associated methicillin-resistant Staphylococcus aureus disease?. J Infect Dis.

[18] Bubeck Wardenburg J, Bae T, Otto M, Deleo FR, Schneewind O (2007). Poring over pores: alpha-hemolysin and Panton-Valentine leukocidin in Staphylococcus aureus pneumonia.. Nat Med.

[19] Fraser C, Hanage WP, Spratt BG (2005). Neutral microepidemic evolution of bacterial pathogens.. Proc Natl Acad Sci U S A.

[20] Aires de Sousa M, Conceicao T, Simas C, de Lencastre H (2005). Comparison of genetic backgrounds of methicillin-resistant and -susceptible Staphylococcus aureus isolates from Portuguese hospitals and the community.. J Clin Microbiol.

[21] Feil EJ, Cooper JE, Grundmann H, Robinson DA, Enright MC (2003). How clonal is Staphylococcus aureus?. J Bacteriol.

[22] Lindsay JA, Moore CE, Day NP, Peacock SJ, Witney AA (2006). Microarrays reveal that each of the ten dominant lineages of Staphylococcus aureus has a unique combination of surface-associated and regulatory genes.. J Bacteriol.

[23] Turner KM, Feil EJ (2007). The secret life of the multilocus sequence type.. Int J Antimicrob Agents.

[24] Kennedy AD, Otto M, Braughton KR, Whitney AR, Chen L (2008). Epidemic community-associated methicillin-resistant Staphylococcus aureus: recent clonal expansion and diversification.. Proc Natl Acad Sci U S A.

[25] Tang CT, Nguyen DT, Ngo TH, Nguyen TM, Le VT (2007). An outbreak of severe infections with community-acquired MRSA carrying the Panton-Valentine leukocidin following vaccination.. PLoS ONE.

[26] Ruimy R, Maiga A, Armand-Lefevre L, Maiga I, Diallo A (2008). The carriage population of Staphylococcus aureus from Mali is composed of a combination of pandemic clones and the divergent Panton-Valentine leukocidin-positive genotype ST152.. J Bacteriol.

[27] Nickerson EK, Wuthiekanun V, Day NP, Chaowagul W, Peacock SJ (2006). Meticillin-resistant Staphylococcus aureus in rural Asia.. Lancet Infect Dis.

[28] Lo WT, Lin WJ, Tseng MH, Lu JJ, Lee SY (2007). Nasal carriage of a single clone of community-acquired methicillin-resistant Staphylococcus aureus among kindergarten attendees in northern Taiwan.. BMC Infect Dis.

[29] Lo WT, Lin WJ, Tseng MH, Wang SR, Chu ML (2008). Risk factors and molecular analysis of panton-valentine leukocidin-positive methicillin-resistant Staphylococcus aureus colonization in healthy children.. Pediatr Infect Dis J.

[30] de Neeling AJ, van den Broek MJ, Spalburg EC, van Santen-Verheuvel MG, Dam-Deisz WD (2007). High prevalence of methicillin resistant Staphylococcus aureus in pigs.. Vet Microbiol.

[31] Sakwinska O, Kuhn G, Balmelli C, Francioli P, Giddey M (2008). Genetic diversity and ecological success of colonizing Staphylococcus aureus.. Appl Environ Microbiol.

[32] Feil EJ, Li BC, Aanensen DM, Hanage WP, Spratt BG (2004). eBURST: inferring patterns of evolutionary descent among clusters of related bacterial genotypes from multilocus sequence typing data.. J Bacteriol.

[33] Sakwinska O, Kuhn G, Balmelli C, Francioli P, Giddey M (2009). Genetic diversity and ecological success of Staphylococcus aureus strains colonizing humans.. Appl Environ Microbiol.

[34] Jolley KA, Kalmusova J, Feil EJ, Gupta S, Musilek M (2000). Carried meningococci in the Czech Republic: a diverse recombining population.. J Clin Microbiol.

[35] Cooper JE, Feil EJ (2006). The phylogeny of Staphylococcus aureus - which genes make the best intra-species markers?. Microbiology.

[36] Yu F, Chen Z, Liu C, Zhang X, Lin X (2008). Prevalence of Staphylococcus aureus carrying Panton-Valentine leukocidin genes among isolates from hospitalised patients in China.. Clin Microbiol Infect.

[37] Nubel U, Roumagnac P, Feldkamp M, Song JH, Ko KS (2008). Frequent emergence and limited geographic dispersal of methicillin-resistant Staphylococcus aureus.. Proc Natl Acad Sci U S A.

[38] Takano T, Higuchi W, Otsuka T, Baranovich T, Enany S (2008). Novel characteristics of community-acquired methicillin-resistant Staphylococcus aureus strains belonging to multilocus sequence type 59 in Taiwan.. Antimicrob Agents Chemother.

[39] Takano T, Saito K, Teng LJ, Yamamoto T (2007). Spread of community-acquired methicillin-resistant Staphylococcus aureus (MRSA) in hospitals in Taipei, Taiwan in 2005, and comparison of its drug resistance with previous hospital-acquired MRSA.. Microbiol Immunol.

[40] Chen CJ, Su LH, Chiu CH, Lin TY, Wong KS (2007). Clinical features and molecular characteristics of invasive community-acquired methicillin-resistant Staphylococcus aureus infections in Taiwanese children.. Diagn Microbiol Infect Dis.

[41] Bauer AW, Kirby WM, Sherris JC, Turck M (1966). Antibiotic susceptibility testing by a standardized single disk method.. Am J Clin Pathol.

[42] Lina G, Piemont Y, Godail-Gamot F, Bes M, Peter MO (1999). Involvement of Panton-Valentine leukocidin-producing Staphylococcus aureus in primary skin infections and pneumonia.. Clin Infect Dis.

[43] Enright MC, Day NP, Davies CE, Peacock SJ, Spratt BG (2000). Multilocus sequence typing for characterization of methicillin-resistant and methicillin-susceptible clones of Staphylococcus aureus.. J Clin Microbiol.

[44] Robinson DA, Enright MC (2004). Multilocus sequence typing and the evolution of methicillin-resistant Staphylococcus aureus.. Clin Microbiol Infect.

[45] Jolley KA, Feil EJ, Chan MS, Maiden MC (2001). Sequence type analysis and recombinational tests (START).. Bioinformatics.

[46] Tamura K, Dudley J, Nei M, Kumar S (2007). MEGA4: Molecular Evolutionary Genetics Analysis (MEGA) software version 4.0.. Mol Biol Evol.

[47] Haubold B, Hudson RR (2000). LIAN 3.0: detecting linkage disequilibrium in multilocus data. Linkage Analysis.. Bioinformatics.

